# Technology-Based Interventions in Substance Use Treatment to Promote Health Equity Among People Who Identify as African American/Black, Hispanic/Latinx, and American Indian/Alaskan Native: Protocol for a Scoping Review

**DOI:** 10.2196/34508

**Published:** 2022-05-17

**Authors:** Emily G Hichborn, Sarah K Moore, Phoebe R Gauthier, Nico O Agosti, Kathleen D Bell, Jesse S Boggis, Chantal A Lambert-Harris, Elizabeth C Saunders, Avery M Turner, Bethany M McLeman, Lisa A Marsch

**Affiliations:** 1 Center for Technology and Behavioral Health Geisel School of Medicine Dartmouth College Lebanon, NH United States; 2 The Dartmouth Institute for Health Policy and Clinical Practice Geisel School of Medicine Dartmouth College Hanover, NH United States

**Keywords:** health disparities, scoping review, social determinants of health, substance use, treatment, technology-based interventions, underrepresented

## Abstract

**Background:**

Technology-based interventions (TBIs; ie, web-based and mobile interventions) have the potential to promote health equity in substance use treatment (SUTx) for underrepresented groups (people who identify as African American/Black, Hispanic/Latinx, and American Indian/Alaskan Native) by removing barriers and increasing access to culturally relevant effective treatments. However, technologies (emergent and more long-standing) may have unintended consequences that could perpetuate health care disparities among people who identify as a member of one of the underrepresented groups. Health care research, and SUTx research specifically, is infrequently conducted with people who identify with these groups as the main focus. Therefore, an improved understanding of the literature at the intersection of SUTx, TBIs, and underrepresented groups is warranted to avoid exacerbating inequities and to promote health equity.

**Objective:**

This study aims to explore peer-reviewed literature (January 2000-March 2021) that includes people who identify as a member of one of the underrepresented groups in SUTx research using TBIs. We further seek to explore whether this subset of research is race/ethnicity conscious (does the research consider members of underrepresented groups beyond their inclusion as study participants in the introduction, methods, results, or discussion).

**Methods:**

Five electronic databases (MEDLINE, Scopus, Cochrane Library, CINAHL, and PsycInfo) were searched to identify SUTx research using TBIs, and studies were screened for eligibility at the title/abstract and full-text levels. Studies were included if their sample comprised of people who identify as a member of one of the underrepresented groups at 50% or more when combined.

**Results:**

Title/abstract and full-text reviews were completed in 2021. These efforts netted a sample of 185 studies that appear to meet inclusionary criteria. Due to the uniqueness of tobacco relative to other substances in the SUTx space, as well as the large number of studies netted, we plan to separately publish a scoping review on tobacco-focused studies that meet all other criteria. Filtering for tobacco-focused studies (n=31) netted a final full-text sample for a main scoping review of 154 studies. The tobacco-focused scoping review manuscript is expected to be submitted for peer review in Spring 2022. The main scoping review data extraction and data validation to confirm the accuracy and consistency of data extraction across records was completed in March 2022. We expect to publish the main scoping review findings by the end of 2022.

**Conclusions:**

Research is needed to increase our understanding of the range and nature of TBIs being used in SUTx research studies with members of underrepresented groups. The planned scoping review will highlight research at this intersection to promote health equity.

**International Registered Report Identifier (IRRID):**

DERR1-10.2196/34508

## Introduction

Pervasive inequalities known as health disparities arise when disease incidence, prevalence, morbidity, mortality, or survival is worse in a population subgroup than in the general population. Health and health care are strongly influenced by race and ethnicity, socioeconomic status (SES), and other characteristics, which can impact access to quality health care. Health disparities have produced negative social and economic consequences on a national scale in the United States [1]. Historically, health treatment intervention research has infrequently been conducted with people who identify as a member of one of the underrepresented groups as a principal focus; this is equally true for the substance use and substance use disorder (SUD) treatment (SUTx) field [2]. A general lack of attention to issues of access, inclusion, retention, equity in outcomes, and culturally relevant research are sources of disparities in substance use (and related consequences) for members of underrepresented groups [3]. Fortunately, there is a small but growing body of literature of best practices for researching diverse groups that may serve as a guide for those motivated to better understand how to reduce health disparities [4] and more equally distribute benefits across populations [5].

Technology-based interventions (TBIs) for SUTx show great promise for expanding reach, providing access to high-quality, personalized, evidence-based treatments (EBTs), while reducing barriers to treatment [6] (eg, access, adoption/uptake, adherence, effectiveness [1], or cultural appropriateness/relevance of TBIs [7]). The rapid proliferation of TBIs has revolutionized clinical and research practices, and these fields will continue to grow rapidly and have a substantial impact on population health [2]. However, there are legitimate concerns that these promising technological advances can lead to unintended consequences such as perpetuating health and health care disparities for people who identify as African American/Black, Hispanic/Latinx, and American Indian/Alaskan Native [8]. Describing common goals of researchers in the health research community when trying to improve well-being, the thinking is that the worst thing that could happen is that our efforts have no effect. However, there is a real and more insidious possibility: that our technological interventions do work but that they work better for those who are already privileged, creating intervention-generated inequalities [1].

People who identify as members of underrepresented groups comprise over one-third of the admissions to publicly funded SUTx programs [9]; however, recent research suggests these individuals may be at particular risk for poor treatment outcomes, due in part to socioeconomic factors [10] and racism [11,12]. Despite socioeconomic challenges, the digital gap among members of underrepresented groups and Caucasian individuals has narrowed over the past 15 years [13]. Technology-based interventions for substance use and SUDs show substantial promise for providing access to high-quality EBT. Increased understanding of the use of TBIs in the field of SUTx with directed attention to how technology may reduce health disparities/promote health equity, or can be harnessed to do so, is warranted.

Given the indications for our literature synthesis—*to identify key characteristics or factors related to a concept* and *to examine how research is conducted on a certain topic*—a scoping review is deemed the most appropriate method [14]. A preliminary search of MEDLINE, the Cochrane Database of Systematic Reviews, and *JBI Evidence Synthesis* was conducted, and no current or underway systematic reviews or scoping reviews on the topic were identified.

This scoping review aims to characterize the range and nature of TBIs being used in SUTx research studies with a majority of people who identify as African American/Black, Hispanic/Latinx, and American Indian/Alaskan Native. Most interventions lack the inclusion of vulnerable/underrepresented populations, which contributes to limited generalizability and the meaningful use of TBIs for improving community health, further perpetuating health disparities [15]. Thus, by identifying and comparing published interventions that *include* such groups, we are taking the first step to interrupt the perpetuation of disparities by attending to access (recruitment/retention); equity in outcomes (evaluations of health changes related to use of the TBI; eg, tobacco cessation); evaluation of the technology itself, such as the usability and helpfulness; and the cultural relevance of the research (multicultural approach vs generalizability approach) [4].

Additionally, we aim to identify and examine the race/ethnicity consciousness of studies included in this review, meaning that if a study considers underrepresented groups, beyond their inclusion as study participants, in the introduction, methods, results, or discussion, it will be considered race/ethnicity conscious (Textbox 1). By taking this further step to explore the extent to which these studies that include members of underrepresented groups are explicit about race or ethnicity and the impacts of TBIs for particular people (because intervention outcomes from one group do not necessarily generalize to other groups [16]), we further highlight research efforts to promote health equity. We believe that by identifying the studies that are race/ethnicity conscious and examining the content of the portions of the manuscript (methods, analytic plan, discussion, or interpretation of results) that meet that criterion, we will be able to provide substantive insight into how people who identify as members of underrepresented groups are included in sociotechnical TBI development, recognizing the interrelatedness of the social and technical factors in particular environments creating optimal tools to enhance well-being [17]. Finally, we plan to summarize findings from race conscious studies that underscore insights that may help other researchers design or adapt designs such that the effects for members of underrepresented groups promote health equity. Importantly, the identification of included studies that are not found to be race conscious may still yield substantial insights and substantive knowledge about how these communities are affected by the use of TBIs for SUTx.

**Race/ethnicity conscious research practices in manuscripts.**

If a study includes reference to race or ethnicity in one or more sections of their manuscript it will be considered race/ethnicity conscious. Examples of race/ethnicity conscious studies are those that:

**Introduction**

- Mention one or more racial or ethnic groups in the literature review or enlist a theory that is described as one that may help address health disparities among racial/ethnic/underrepresented groups. An example of this includes sociological trust theory—bridge between broad lens of culturally informed design and attention to trust or distrust.

**Methods**

- Plan for health equity focused analyses by powering studies for subgroup analyses or analyses of effect modifiers while following rigorous standards for heterogeneity of treatment effect analyses or use race or ethnicity as a covariate in analyses, or in some other way consider race in the plan for analyzing the data. Race conscious methods may also include references to recruitment or retention efforts aimed at racial or ethnic groups such as cultural tailoring of materials or consideration of matching staff race/ethnicity to that of the sample participants.

**Results**

- Present findings in a way that highlights differences/similarities for members of different racial and/or ethnic groups

**Discussion**

- Interprets findings for members of racial or ethnic groups by locating results in the context of other development or treatment literature

## Methods

### Scoping Review

This review will adhere to the PRISMA-P (Preferred Reporting Items for Systematic Reviews and Meta-Analysis Protocols) guidelines, including search strategy, selection criteria, data extraction, and analysis [18]. Additionally, the review will be conducted using the Arksey and O’Malley [19] methodological framework for scoping reviews that recommends the following six steps: (1) identifying the research question; (2) identifying relevant studies; (3) study selection; (4) charting the data; (5) collating, summarizing, and reporting results; and (6) consultation. The review will be reported according to PRISMA-ScR (PRISMA Extension for Scoping Reviews) guidelines [20]. This scoping review was initiated in January 2021 and is expected to be completed by the end of 2022.

### Step 1: Identifying the Research Question

The following research question was identified to guide the scoping review: Does the use of digital TBIs in SUTx research promote health equity among people who identify as African American/Black, Hispanic/Latinx, and American Indian/Alaskan Native?

To increase clarity and focus needed to inform subsequent phases of the research process, we operationally defined health equity as the inclusion of members of underrepresented groups (previously specified) in research of TBIs for substance use, as well as the extent to which the research is race/ethnicity conscious. Thus, the following questions are intended to establish an effective search strategy [16]:

- Does research enlisting TBIs in SUTx include people who identify as African American/Black, Hispanic/Latinx, or American Indian/Alaskan Native?
- If the substance use research enlisting TBIs does include people who identify as African American/Black, Hispanic/Latinx, or American Indian/Alaskan Native, is it race/ethnicity conscious?

#### Population

The focus of this review is on the following racial and ethnic groups: African American/Black, Hispanic/Latinx, and American Indian/Alaskan Native. The former two groups are included as they are the largest racial and ethnic groups underrepresented in SUTx in the United States [21]. People who identify as African American/Black and Hispanic/Latinx have been found to be less likely to complete treatment compared to Caucasian individuals [22,23] for a variety of reasons such as disproportionately lower SES [10], greater likelihood of incarceration [24], lower perceived treatment efficacy, and cultural factors [25]. The review also focuses on people who identify as American Indian/Alaskan Native, as members of this group of Americans have the highest rates of substance use problems compared with members of other groups, and access to care remains extremely limited [26,27].

#### Concept

Technology-based interventions are used as an umbrella term that encompasses interventions such as mobile-based interventions, computer-based interventions, and web-based interventions. The term TBIs was selected because it encompasses a broad array of platforms, including the computer, internet, social media, and mobile apps [28].

#### Context

Eligible papers describe the use of TBIs that facilitate the inclusion of members of underrepresented groups in SUTx or support delivery of SUTx to members of underrepresented groups. Examples of potential TBIs used to support SUTx are interactive voice response (IVR) and ecological momentary assessments. Substance use treatment defined for this review includes treatment for any type of SUD, including alcohol, tobacco, and other drugs; treatment for substance use that does not meet disorder criteria yet is still considered risky, problematic, or heavy use; treatment provided in any type of setting (inpatient or outpatient settings); treatment provided to both individuals and people in group settings; and treatments that are both evidence-based with rich supporting literature (ie, medication-assisted treatments including pharmacotherapies such as naltrexone, methadone, buprenorphine), behavioral or psychological therapies (brief interventions, cognitive behavioral therapy, contingency management, drug counseling, motivational interviewing), and integrated psychotherapy and pharmacotherapy, as well as newly designed treatments that are being presented in pilot/efficacy studies. Importantly, assessment, or the process of obtaining information about a participant’s drug use and how it affects their life, is considered an integral part of treatment, and therefore, studies that focus on assessment or monitoring impacts of use with a focus on reduced use or cessation/abstinence are also included.

### Step 2: Identifying Relevant Studies

#### Information Sources and Search Strategy

Before enlisting the help of Dartmouth College research librarians, study team members conducted preliminary independent literature searches in PubMed and Google Scholar using search terms associated with the three domains of interest: TBIs, SUTx, and sample inclusion of members of underrepresented groups. This step netted dozens of research studies that appeared to meet the inclusion criteria. Further, we reviewed the reference lists of these studies (examining titles and abstracts) for additional literature, identifying many additional studies that appeared to meet our inclusion criteria. Based on this preliminary literature search, we believed there would be ample evidence that met our search criteria and therefore elected to only include US-based peer-reviewed studies. Further underscoring the rationale for limiting our focus on US studies, underrepresented groups in the United States differ from underrepresented groups in other countries.

The search strategy aimed to locate published peer-reviewed studies based on the preliminary data analysis. An initial limited search of MEDLINE was undertaken to identify studies that met selected criteria. Search terms were organized by two of the domains of interest: TBIs and SUTx. However, the target race and ethnicity demographics were not included in this search strategy. Rather, team members manually reviewed for this criterion at the title/abstract and full-text level because the wide variation in search terms used to identify sample makeup based on race and ethnicity may inadvertently eliminate eligible studies.

The text contained in the titles and abstracts of relevant studies, and the index terms used to describe the studies, was used to develop a full search strategy for MEDLINE, Scopus, Cochrane Library, CINAHL, and PsycInfo (see Multimedia Appendix 1). The search strategy, including all identified keywords and index terms, was adapted for each included database or information source.

This scoping review was conducted by nine researchers from a variety of disciplines at Dartmouth College. This larger group was split into three smaller clusters to complete blinded decisions on each study at the title/abstract and full-text review phases. Additionally, these groups conducted data extractions from each included study.

### Step 3: Study Selection

#### Types of Sources

We considered peer-reviewed, qualitative, quantitative, and mixed methods studies. Study designs include but are not limited to randomized trials, randomized controlled trials (efficacy/effectiveness), feasibility/acceptability pilots, formative development, secondary analyses (eg, mechanisms or moderators), and assessments.

#### Eligibility Criteria

Eligible studies are US-based, English language, peer-reviewed, published between January 2000 and March 2021, include participants 12 years and older (average age of onset of substance use), and 50% or more of the sample represents individuals who identify as African American/Black, Hispanic/Latinx, or American Indian/Alaskan Native when combined. While most people who identify as Hispanic/Latinx also identify as White/Caucasian, identification as Hispanic/Latinx is the prioritized category and takes precedence over race given that most studies do not report race stratified by ethnicity. When calculating the 50%, we do not include categories of *other* or *multi-race*, as they are too vague to help address the scoping review’s aims. We chose this 50% threshold after reviewing the guidance of a research review on EBT for ethnic minority youth. To evaluate treatments for ethnic minority youth, Huey and Polo [29] suggested that an intervention could be considered well-established, probably efficacious, or possibly efficacious for ethnic minority youth if supporting studies met one or more of three conditions: (1) at least 75% of participants were ethnic minorities, (2) if either separate analyses with the subset of ethnic minority participants demonstrated superiority of treatment over control/comparison conditions, or (3) analyses showed ethnicity did not statistically moderate treatment outcomes. We decided to use 50% to be more inclusive and capture additional studies. Additionally, eligible studies include a TBI integrated into SUTx.

#### Exclusion Criteria

Studies were excluded if they are conducted outside of the United States or published outside of the study window (January 2000 to March 2021). Furthermore, we removed protocol papers, as well as papers that detail the work researchers plan to perform in the future. Other papers that are not included in the final list are reviews, commentaries, editorials and opinion pieces, student theses, book chapters, and guidelines. Additionally, we excluded studies solely focused on mental health, pharmacological, cost evaluations, telephone counseling (eg, tobacco quitlines), and primary prevention interventions.

#### Screening and Selection Procedure

Following the search, all netted citations were uploaded by the research librarians into Endnote X9 (Clarivate Analytics), a citation management software program, to manage references and remove duplicates [30]. To facilitate study screening and selection, all citations obtained using the search strategy were imported into Rayyan*,* a web-based tool used to assist researchers in screening, selecting, and labeling studies for systematic reviews [31]. Rayyan was used to blind individual reviewers on each of the three teams to individual team member’s decisions regarding inclusion/exclusion of each study. This process was used at both the title/abstract and full-text review level. Once citations were mutually agreed upon by team members and therefore included, potentially eligible studies were retrieved in full and their citation details imported into Rayyan. The full text of selected citations was assessed in detail against the inclusion criteria by two or more independent reviewers on each of the three teams. Reasons for exclusion of sources at the full text stage will be recorded and reported in the scoping review. Examples of this include international studies, studies that did not meet sample criteria, and wrong publication type. Any disagreements that arose between the reviewers at each stage of the selection process were resolved through discussion or with additional reviewers.

### Step 4: Charting the Data

#### Data Extraction

Data were extracted from included studies by our team of nine independent reviewers using a standardized data extraction tool built into Excel (Microsoft Corporation; see Multimedia Appendix 2) over approximately 6 months. We extracted basic study information including first author, year of publication, and study aims/purpose. In addition, we extracted population, design, sample (racial and ethnic profile), details related to the TBI, substances that are the focus of the TBI, main outcomes, and key findings that relate to the scoping review questions regarding race and ethnicity consciousness. Reviewers were divided into three teams to pilot the extraction form on three studies. The entire nine-person team met to discuss issues arising during the pilot experience, refine procedures, and revise the form. The extraction process was iteratively modified as necessary throughout the conduct of the scoping review and will be detailed in the scoping review outcomes paper. Any disagreements that arose between the reviewers were resolved through discussion or with additional reviewers. Throughout the data extraction process, methodical quality checks were conducted to ensure the consistency, accuracy, and thoroughness of the information extracted. In one instance, the authors of a research program that included several studies eligible for our review were contacted to request additional data.

### Step 5: Collating, Summarizing, and Reporting the Results

#### Data Analysis and Presentation

Evidence presented in our review will directly respond to the review objectives and questions, and will be presented in tabular form. First, we will characterize the included studies by author, year of publication, aims, design, sample, population, and substance. Second, we will characterize the TBIs being used in SUTx research with members of underrepresented groups. Third, we will examine how SUTx research with people who identify as African American/Black, Hispanic/Latinx, and American Indian/Alaskan Native using TBIs is being conducted, highlighting studies that exemplify race/ethnicity conscious research practices throughout their published manuscripts that may promote health equity. We will also provide a narrative summary of the tabulated results and describe how the results relate to the review’s questions and aims.

### Step 6: Consultation

No patients were involved in the design of this scoping review. Experts with experience conducting scoping reviews as well as conducting research using TBIs with underrepresented groups may be consulted in the presentation of findings for this scoping review.

## Results

The title and abstract review was completed in 2021, netting 6897 articles. Following the exclusion of 5615 records not meeting study inclusion criteria, a full-text review was conducted on 1158 records over a 3-month period. Following the exclusion of 935 full-text records for four primary reasons—race/ethnic criteria not met (n=486), wrong publication type (eg, conference abstract; n=181), not a US-based study (n=161), or not a TBI (n=89)—185 records remained. We plan to publish the full PRISMA diagram with the main scoping review. Notably, due to the uniqueness of tobacco relative to other substances in the SUTx space [32], as well as the large number of studies netted, our team made the decision to separately publish a scoping review on tobacco-focused studies that meet all other criteria. Filtering for tobacco-focused studies (n=31) netted a final full-text sample for a main scoping review of 154 studies. The tobacco-focused scoping review manuscript is expected to be submitted for peer review in spring 2022. The main scoping review data extraction is completed. By March 2022, data validation to confirm the accuracy and consistency of data extraction across records will be completed, and we expect to publish the main scoping review findings by the end of 2022.

## Discussion

### Potential Impact and Future Directions

The proposed scoping review will have the potential to provide a status update on TBIs for SUTx and their potential to promote health equity among people from historically underrepresented groups. By identifying the universe of US-based studies on TBIs for SUTx that include people who identify as African American/Black, Hispanic/Latinx, and American Indian/Alaskan Native in the past 20 years, this review will likely provide guidance on how researchers and developers may avoid worsening inequities through increasing the race/ethnicity consciousness of both research practices and technological innovations. By identifying research on TBI use in SUTx that include members of underrepresented groups, we anticipate the main findings to include insights into issues of participant access to research on TBIs (eg, the prevalence of one group’s representation in the netted review’s sample compared with that of others highlighting research gaps, recruitment and retention strategies, incentive structures, or platform access descriptions), the main types of TBIs being used with members of these groups (eg, IVR, SMS text messages, computer-delivered, smartphone, social media, or virtual reality), the cultural relevance or lack thereof (end-user engagement) of the TBIs under study, the relative efficacy of certain TBIs versus comparators, and an in-depth understanding of the extent of the race consciousness of the netted sample of studies included in the review. While there is no prior work on this specific niche of the literature, several other published scoping reviews at the intersection of TBIs and health inequities may be of interest to readers [33,34].

As the landscape of SUTx evolves and as underrepresented groups grow (eg, migration) in the United States, this review may become more pertinent for TBI SUTx researchers in particular. The results of the search and the study inclusion process will be reported in full in the final scoping review, published in a peer-reviewed journal, and presented in a PRISMA-ScR flow diagram [35]. This protocol is designed to highlight our methods, facilitate replication, alert researchers to the fact that the review is being conducted [14,35], and forecast anticipated findings.

### Limitations

By limiting inclusion in the scoping review to studies with 50% of the sample comprised of the three race/ethnicity categories, we recognize that we may unnecessarily limit the sample of studies for inclusion in the scoping review. However, we chose this threshold after reviewing the guidance of an article that summarized research on EBT for ethnic minority youth that suggested that an intervention could be considered possibly efficacious for ethnic minority youth if at least 75% of participants were ethnic minorities [29]. In choosing the less conservative number of 50%, we planned to be more inclusive. Relatedly, by limiting our inclusion criteria to studies with 50% of the sample comprised of the three race/ethnicity categories, we exclude studies that may have findings for mixed race or ethnicities. It has also come to our attention that it might have been useful to consider studies where the initial recruitment included a substantial proportion of participants who identified as one of the underrepresented groups but not necessarily in the final sample. Inclusion of such studies might have helped to identify shortcomings in study design that inadvertently exclude these individuals that would have contributed to the scoping review’s goal to inform equity impacts of TBIs. We also recognize that limiting our scoping review to the English language and only US studies is common in systematic reviews and could result in biased interpretation of findings [36]. However, the research team lacks fluency in additional languages, limiting our ability to conduct a review beyond an English-only data set. A suggestion for future research will include a recommendation to broaden the language criteria beyond English, particularly to include Spanish and Native language studies given the focus of this review on the impact of TBIs for SUTx for individuals who identify as African American/Black, Hispanic/Latinx, and Native American/Alaskan Native. Additionally, while we will use several databases, there is the possibility that we have overlooked some relevant research that meets our search criteria.

## Appendix

Multimedia Appendix 1 Medline (Ovid). Database(s): Ovid MEDLINE and Epub Ahead of Print, In-Process, In-Data-Review, and Other Non-Indexed Citations and Daily 1946 to March 29, 2021.

Multimedia Appendix 2 Data extraction items.

## Abbreviations

EBT: evidence-based treatment
IVR: interactive voice response
PRISMA-P: Preferred Reporting Items for Systematic Reviews and Meta-Analyses Protocols
PRISMA-ScR: Preferred Reporting Items for Systematic Reviews and Meta-Analyses Extension for Scoping Reviews
SES: socioeconomic status
SUD: substance use disorder
SUTx: substance use treatment
TBI: technology-based intervention
