# Supplementary material for: Technology-Based Interventions in Substance Use Treatment to Promote Health Equity Among People Who Identify as African American/Black, Hispanic/Latinx, and American Indian/Alaskan Native: Protocol for a Scoping Review
Source: JMIR Res Protoc. 2022 May 17;11(5):e34508. doi: 10.2196/34508 (PMC9157317; doi:10.2196/34508)
Supplement: Multimedia Appendix 1 [file resprot_v11i5e34508_app1.docx]

| **#** | **Searches** | **Results** |
| --- | --- | --- |
| 1 | exp *Cell Phone/ or *Fitness Trackers/ or exp *Mobile Applications/ or exp *Therapy, Computer-Assisted/ or *Internet-Based Intervention/ or exp *Computers, Handheld/ or *Social Media/ or exp *Telemedicine/ or exp *Virtual Reality Exposure Therapy/ or *Virtual Reality/ or *Patient Portals/ or ((delivered or app or application* or approach* or therap* or intervention* or treatment*) adj5 (Technology or Mobile or Internet or Website or Online or Web or Computer or Laptop or Smartphone* or "Smart phone*" or cellular or Ipad or Ipod or "PORtable media player" or "Mp3 player" or "Mp4 player" or Tablet or "Hand held device" or "Personal digital assistant" or "Virtual reality" or Electronic or "Messaging system" or "Text messag*" or Skype or Zoom or Webex or Video or DVD or "smart watch*" or "apple watch" or Garmin or fitbit or wearable* or "digital camera" or "gaming console*" or xbox or playstation or PS4 or PS5 or wii or "nintendo" or "digital assistant" or "smart speaker*" or siri or "google assistant" or Alexa or email or "social media" or facebook or instagram or twitter or youtube)).ti,ab,kf. or ("E-health" or Ehealth or "M-health" or Mhealth or "Mobile health" or Telemedicine or Telehealth or Telepsychiatry or "Interactive voice response" or "digital therapeutic*" or "digital health").ti,ab,kf. | 208406 |
| 2 | exp *alcohol drinking/ or exp *substance-related disorders/ or *"diagnosis, dual (psychiatry)"/ or exp *Illicit Drugs/ or *designer drugs/ or (Alcoholism or ((alcohol or binge) adj2 drinking)).ti,ab,kf. or ((alcohol or Tobacco or marijuana or cannabis or narcotic or narcotics or opioid* or opiate or morphine or heroin or methadone or oxycodone or oxycontin or buprenorphine or suboxone or vicodin or hydrocodone or cocaine or LSD or amphetamine* or inhalant* or PCP or phencyclidine or methamphetamine* or spice or "bath salts" or K2 or MDMA or psychostimulant* or "Street drug*" or drug* or substance) adj3 (abus* or addict* or dependen* or misuse or "use disorder*" or using)).ti,ab,kf. | 384692 |
| 3 | 1 and 2 | 3674 |
| 4 | limit 3 to (english language and yr="2000 -Current") | 3494 |
