# Supplementary material for: Technology-Based Interventions in Substance Use Treatment to Promote Health Equity Among People Who Identify as African American/Black, Hispanic/Latinx, and American Indian/Alaskan Native: Protocol for a Scoping Review
Source: JMIR Res Protoc. 2022 May 17;11(5):e34508. doi: 10.2196/34508 (PMC9157317; doi:10.2196/34508)
Supplement: Multimedia Appendix 2 [file resprot_v11i5e34508_app2.docx]

| **First Author Last Name** |
| --- |
| **Year of Study** |
| **Date of Extraction** |
| **Extracted by** |
| **Study Aims:** Description of the goals, aims, objectives, purpose, of the study. |
| **Design:** randomized trials, and randomized control trials (efficacy/effectiveness), feasibility/acceptability/usability, feasibility/efficacy or effectiveness, feasibility/mixed methods, development/formative, development/feasibility/acceptability, development/implementation, secondary analysis (e.g. mechanisms or moderators), assessment, and other. |
| **Population:** Description of the target population the study intends to enroll.  **Digital Literacy Required:** Yes/No |
| **Number of Participants** |
| **Sample Summary:** Description of the participants actually enrolled, including gender and racial/ethnic breakdown only. |
| **African American/Black:** Yes/No |
| **Hispanic/Latino:** Yes/No |
| **American Indian/Alaska Native:** Yes/No |
| **Caucasian:** Yes/No |
| **Asian:** Yes/No |
| **Native Hawaiian/Pacific Islander:** Yes/No |
| **More Than One Race:** Yes/No |
| **Recruitment Plan:** Description of how candidates are informed of the study and introduced to study. |
| **Retention Plan:** Description of any efforts to retain participants in research study. |
| **Context:** Setting where the intervention is taking place and other relevant details (urban, rural, remote). If setting is not adequately described, use name of city/town/geographical location. |
| **Primary SUD:** Substance or substance use disorder targeted by TBI. |
| **SUD, Other or Multiple:** complete if there are multiple primary substances or substance. |
| **TBI Description and Name** |
| **Device:** Delivery device for intervention. |
| **Other Device** |
| **Delivery Mode + Intensity/frequency:** Description of how the intervention is delivered to participant including the intensity and duration. |
| **Was TBI combined with other intervention/TAU:** Interventional components, for example, contingency management, behavioral therapy, supportive messages. |
| **TBI Combined with Other Intervention:** Treatment comparator.  **TBI Behavior Change Theory and Techniques:** The underlying theory of the TBI or model and techniques used as the basis for persuasive system. |
| **TBI Developmental Framework or Model:** Model guiding design/development of the TBI. |
| **End-User Involvement:** Description of methods used to involve/engage end-users in development/design, and/or adaptation of TBI, opportunity for end-user to provide feedback on TBI to inform acceptability, etc. (intervention focused outcomes). |
| **Main Outcomes:** Patient focused (health changes related to tech, perceived value of intervention) and/or Implementation/Tech focused (comparison of electronic to paper records). Description of main outcomes as they relate to either impact for participant or understanding/optimization of TBI. |
|  |
| **Author Recommendations:** The authors’ conclusions, recommendations on the TBI and associated outcomes for underrepresented groups. |
| **Race/Ethnicity Conscious:** If a study includes reference to race and ethnicity in one or more sections of their manuscript (i.e. Introduction, Methods, Results and/or Discussion). |
| **Other/Comments** |
